# Allergic symptoms and sensitisation in adolescents with cows' milk allergy and atopic eczema in infancy

**DOI:** 10.1002/iid3.324

**Published:** 2020-06-21

**Authors:** Sonja Piippo, Mirva Viljanen, Erkki Savilahti, Mikael Kuitunen

**Affiliations:** ^1^ Department of Pediatrics, Children's Hospital, Pediatric Research Center Helsinki University Hospital, University of Helsinki Helsinki Finland

**Keywords:** adolescent, allergic rhinitis, asthma, atopic eczema, cows' milk allergy, specific IgE

## Abstract

**Background:**

The association between atopic sensitisation, atopic eczema (AE) and asthma is known, but distinct roles of allergies on long‐term health are unestablished.

**Objective:**

Evaluation of allergic symptoms and sensitisation in adolescents who in infancy had AE and verified cows' milk allergy (CMA) or AE and a negative CMA challenge, and controls.

**Methods:**

Children with AE, with and without CMA, from a randomised controlled study in 1999‐2001 examining the effect of probiotics on AE severity at older than 12 months of age, attended a follow‐up visit at age 16 to 18, with age‐matched controls. Data came from a questionnaire (ISAAC questionnaire), analysis of serum antigen‐specific immunoglobulin Es (IgEs), and clinical evaluation. Group comparisons were carried out (*χ* ^2^ tests and logistic regression).

**Results:**

Fifty‐two patients with AE and CMA (AE/CMA+ group), 52 with AE and suspicion of CMA (AE/CMA− group), and 57 controls attended a study visit. IgE‐mediated sensitisation was significantly more prevalent in the AE/CMA+ group vs the controls, for horse, cat, dog, egg white and wheat (*P* < .024 for all). For birch, timothy and mugwort (*P* < .008 for all), sensitisation was more prevalent in both the AE/CMA+ group and the AE/CMA− group vs controls. On the basis of questionnaire data the AE/CMA + group reported a significantly higher lifetime prevalence of wheezing (64% vs 35% and 32%; *P* = .001), noninfectious rhinitis (85% vs 62% and 56%; *P* = .004), and hay fever (77% vs 52% and 33%; *P* < .001) vs the AE/CMA− group and the control group, respectively.

**Conclusion and Clinical Relevance:**

Patients with AE and CMA in infancy, as opposed to patients with AE only, or controls, report more allergic symptoms and exhibit more allergic sensitisation in adolescence. This indicates that CMA in infancy is an independent risk factor of allergic disease in adolescence.

## Graphical abstract

We examined the effect of cows' milk allergy (CMA), confirmed by double‐blind, placebo‐controlled food challenge in infancy, on allergic symptoms later in life. These patients reported in adolescence a significantly higher lifetime prevalence of wheeze, noninfectious rhinitis and hay fever, compared to patients with atopic eczema (AE) and a previous suspicion of CMA, and controls. These patients were also more sensitised to all tested allergens, the difference was significant compared to controls.

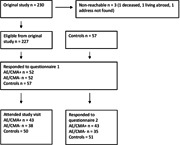

## 1 INTRODUCTION

During recent decades, the prevalence of allergic sensitisation and diseases in industrialised countries has increased.^1^ Major differences in sensitisation patterns exist between countries, with, for example, cows' milk allergy (CMA) being the most common food allergy in early childhood in the western world, affecting 0.5% to 3% of children.^2^, ^3^, ^4^ Studies are, however, difficult to compare, as diagnostic criteria for CMA differ. Some investigators base their diagnoses on immunoglobulin E (IgE)‐mediated sensitisation, others depend on open food challenges, while the gold standard for diagnosis is the double‐blind, placebo‐controlled food challenge (DBPCFC),^5^ which is cumbersome and costly, and hence less used. No immunological markers can predict the development of tolerance accurately. This development, however, is generally favourable, as the majority of cases of CMA resolve spontaneously, with the rate and timing of resolution in studies showing wide variety.^4^, ^6^ Compared to controls, patients with atopic eczema (AE) more frequently develop food allergies (FA). A study found among AE patients with mostly mild to moderate severity of disease, that the incidence of FA was 15.9%.^7^ Several studies have reported a 30% prevalence of FA among children with AE. These studies, however, mostly included children with moderate to severe AE.^8^

Sensitisation to food allergens predispose individuals to later allergic disease, but specific allergen‐associated risks are as yet unestablished. One study revealed that sensitisation to hens' eggs or cows' milk at an early age predicted asthma and rhinoconjunctivitis at 14 years of age, compared with no sensitisation at an early age. In this study, however, the effect was particularly attributed to sensitisation to hens' eggs.^9^ Allergic symptoms and disease undergo transformations over time; most early‐life food allergies resolve, whereas allergic rhinoconjunctivitis and allergic asthma become more prevalent with age. The timing of IgE‐mediated sensitisation tends to follow the allergen order of exposure. In Finland, sensitisation to cows' milk primarily appears in infants as they are exposed to cows' milk protein in formula typically from 3 to 5 months of age. Cows' milk is followed by sensitisation to wheat, fish and egg, still followed by nuts, as they are introduced later in life, according to national recommendations. Food sensitisation is succeeded by sensitisation to outdoor allergens, primarily birch, then timothy and mugwort. Sensitisation to indoor allergens such as furry pets depend on the timing and level of exposure. Sensitisation to food allergens occurs transcutaneously as well as orally, whereas to indoor and outdoor allergens it occurs mainly via inhalation.^10^, ^11^, ^12^ It is unclear whether AE and FA are manifestations of the same atopic disposition or if they represent separate entities that are independent risk factors of subsequent development of allergic rhinitis and asthma.^13^

Most studies on the associations between CMA and other atopic conditions have been focused on infancy and early childhood, and only a few have concerned adolescents or adults. One retrospective study on 807 patients with IgE‐mediated CMA included some patients up to the age of 23. However, the median age at the first visit was 13 months, and the median duration of follow‐up was 54 months. This study revealed a relatively high prevalence of asthma (49%) and allergic rhinitis (40%) in the total study population, which could be explained by the fact that the patients were recruited from a tertiary care centre.^6^ Another study, involving 139 CMA patients, both IgE‐mediated and non–IgE‐mediated, showed development of asthma in 32%, rhinoconjunctivits in 20%, and other FAs in 19%. The patients were aged 14 months to 18 years at the end of the study, and the mean follow‐up time was 7 years.^14^ The role of CMA as an independent risk factor of other allergic conditions remains unclear. The aim of this study was, thus, to assess the implications of early‐life CMA and AE in adolescence.

## 2 METHODS

### 2.1 Study population

For this follow‐up study, the participants were from a randomised controlled study conducted in 1999‐2002 concerning the effect of probiotics on AE.^15^ In brief, patients with AE born in 1999‐2001 that had been referred to a tertiary hospital in connection with a suspicion of CMA at an age below 12 months were recruited. The mean age of the patients in the original study was 6.4 months upon entry. The patients were randomised in a double‐blind manner to receive for 4 weeks either *Lactobacillus rhamnosus* GG, a mix of four probiotics (*L. rhamnosus* GG, *L. rhamnosus* LC705, *Bifidobacterium breve* and *Propionibacterium freudenreichii* ssp. shermanii JS) or placebo. Among these 230 patients, 120 were diagnosed with CMA on the basis of the results of a DBPCFC that was performed 4 weeks after the probiotic intervention. For this follow‐up study recruitment of unselected control patients born in 1999‐2001 was through an advertisement on social media and distribution of leaflets with information on participation at upper secondary schools and vocational schools in Helsinki. Patients were recruited and examined in 2017.

### 2.2 Questionnaires

Upon entry the patients answered the first questionnaire. This questionnaire was designed to assess allergic symptoms (wheeze, rhinitis and rash), doctor‐diagnosed allergic diseases, other primary diseases, contact with agriculture and pets, use of probiotics, and smoking. The second questionnaire concerned the living environment, family, education, diet, and parental medical history. Assessment of allergic symptoms was based on the Finnish version of the International Study of Asthma and Allergies in Childhood (ISAAC) Phase Three Core Questionnaire for children aged 13 to 14 years.^16^, ^17^ The primary outcome was the prevalence of allergic symptoms. For these analyses the response to the question “How many attacks of wheezing have you had in the past 12 months?” was dichotomised as either at least one attack or no attacks. The response to the question “In the past 12 months, have you had a dry cough at night, apart from cough associated with a cold or chest infection?” was also dichotomised as either not at all or at least once.

We used combinations of answers from the ISAAC questionnaire to establish the presence of allergic disease in the previous 12 months. Affirmative answers to the following two questions yielded a diagnosis of current asthma: “Have you had wheezing or whistling in the chest in the past 12 months?” and “Have you ever had asthma?”. Furthermore, affirmative answers to the following three questions yielded a diagnosis of current allergic rhinitis: “Have you ever had a problem with sneezing, or a runny or blocked nose when you DID NOT have a cold or the flu?”, “In the past 12 months, have you had a problem with sneezing, or a runny or blocked nose when you DID NOT have a cold or the flu?” and “In the past 12 months, has this nose problem been accompanied by itchy‐watery eyes?”. Affirmative answers to the following three questions yielded a diagnosis of current eczema: “Have you ever had an itchy rash which was coming and going for at least 6 months?”, “Have you had this itchy rash at any time in the past 12 months?” and “Has this itchy rash at any time affected any of the following places: the folds of the elbows, behind the knees, in front of the ankles, under the buttocks, or around the neck, ears or eyes?” Our study is not part of the ISAAC collaboration.

The presence of doctor‐diagnosed allergic diseases (food allergy, AE, asthma, rhinoconjunctivitis) was based on patient reporting. All patients from the original study were considered to have AE and those diagnosed with CMA were considered to have food allergy in our analyses.

### 2.3 Laboratory measurements

Blood samples were collected at the study visit. Levels of IgEs against specific food allergens (egg white, wheat, milk) and aeroallergens (horse, cat dander, dog dander, timothy grass, mugwort, birch) were measured by using ImmunoCAP reagents (Thermo Fisher Scientific Inc., Waltham, MA).

### 2.4 Clinical evaluation and pulmonary function testing

Patients that had answered the first questionnaire were invited to a study visit. At this visit, they signed a written informed consent document and were examined by a doctor (SP). The severity of AE was evaluated by using the SCORAD calculator.^18^ We performed flow‐volume spirometry to assess lung function by using Medikro Pro equipment (Medikro Oy, Kuopio, Finland). Measurements were both pre‐ and post‐bronchodilation, which was attained by administering salbutamol (0.4 mg). The following spirometry values were analysed: forced vital capacity (FVC), forced expiratory volume in one second (FEV1) and FEV1/FVC. Analysis and reporting of results were based on percentages of predicted values adjusted for age, sex and height.

### 2.5 Statistical analysis

Categorial variables were compared by using the Pearson *χ* ^2^ test. Fisher's exact test was applied when the expected cell count was below five. Significant associations were further analysed by using an adjusted logistic regression model. Continuous variables were analysed by using the independent samples *t* test, the Mann‐Whitney *U* test, one‐way analysis of variance (ANOVA), analysis of covariance (ANCOVA) and the Kruskal‐Wallis test.

An exploratory analysis regarding possible confounders related to ISAAC questionnaire answers was performed by using the *χ* ^2^ test. Analysed factors were sex, siblings (yes/no), patient's age, body mass index, age of mother, age of father, the patient's education, smoking, passive smoking, household pets and use of probiotics during the past 3 years as well as the past 12 months. Confounders included in the final model were sex, pets and passive smoking.

For a separate analysis of original patients only, possible confounders that were analysed were: sex, siblings, parental atopy, siblings' atopy, parental smoking, duration of exclusive breastfeeding, duration of overall breastfeeding, mode of delivery, sensitisation defined as positivity of prick tests (egg white, milk, wheat, any) and/or specific IgEs (milk, wheat), age at introduction of solids, and original intervention with probiotics. Confounders included in the final model were sex, parental atopy and sensitisation to any measured allergen. The results of logistic regression models are presented as odds ratios and adjusted odds ratios (aOR) with 95% confidence intervals (CIs).

Flow‐volume spirometry was analysed by ANCOVA. We identified potential covariates on the basis of factors known to influence spirometry results (flu in the past 2 weeks, cooperation, previous spirometry, age, smoking, education, height, asthma, rhinoconjunctivitis). Among them, asthma and cooperation significantly affected our spirometry values. These covariates were tested in connection with assumptions of normality, linearity, homogeneity of variances, homogeneity of regression slopes, and reliable measurement of the covariate. Only cooperation qualified as a covariate for FVC and FEV1. FEV1/FVC was analysed by way of one‐way ANOVA as no covariates met the above assumptions.

Differences in SCORAD points between groups were compared by using the Kruskal‐Wallis test, as the data was not normally distributed.

We analysed IgE‐mediated sensitisation by using two different cut‐off values: >0.35 and >0.7 kU/L. Differences between groups were analysed by using the *χ* ^2^ test.

For all statistical tests significance was set at *P* < .05. The data was analysed using SPSS software for Windows, version 22 (IBM Corp., Armonk, NY). Bonferroni correction was used to adjust for multiple comparisons. The study was approved by the ethics committee of Helsinki University Hospital for Children and Adolescents. The study complies to the STROBE guidelines.

## 3 RESULTS

### 3.1 Study population

**Figure 1:**
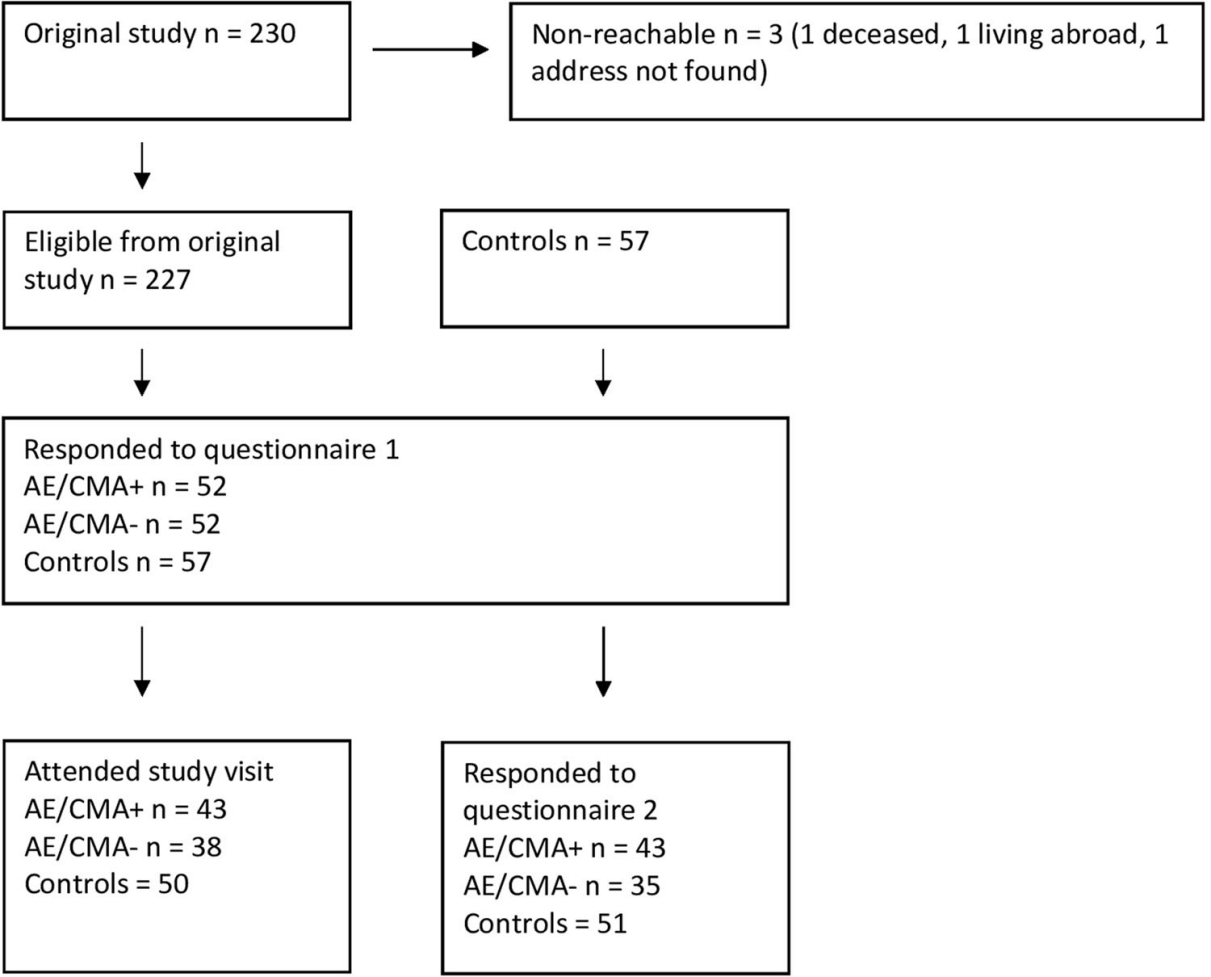
Flowchart of the study

Among 227 patients from the original study, 104 consented to take part in our follow‐up study (Figure 1). Demographic characteristics among all three groups were similar, except for sex, as 84.2% of the control patients were female (Table 1). There were no significant differences between the AE/CMA+ and AE/CMA− groups regarding original probiotic intervention, maternal atopy, paternal atopy, biparental atopy, paternal smoking, maternal smoking during pregnancy, having pets at the time of the original study, or mode of delivery. Neither was there a difference within the AE/CMA+ group regarding CMA diagnosis or sensitisation to milk (measured by either prick test or s‐IgE) (data not shown). However, among patients who had a mother that smoked at the time of the original study, loss to follow‐up was more common compared with those who did not (73.5% and 51.3%, respectively). Loss to follow‐up was also more common in males compared with females (62.2% and 41.9%, respectively).

**Table 1:** Background characteristics of the study population

	AE/CMA+		AE/CMA−		Controls		
	n	%	n	%	n	%	*P* value
Female	25/52	48.1	25/52	48.1	48/57	84.2	**<.001**
Mother's age at birth, mean, y	30.6	NA	31.6	NA	30.4	NA	.541^a^
Maternal atopy	8/51	15.7	5/51	9.8	5/56	8.9	.545
Paternal atopy	4/51	7.8	6/51	11.8	7/56	12.5	.808
Any parent atopic	11/51	21.6	9/51	17.6	9/56	16.1	.815
Both parents atopic	1/51	2.0	2/51	3.9	3/56	5.4	.872
Siblings	38/43	88.4	32/35	91.4	47/51	92.2	.866
Age, mean, y	17.2	NA	17.2	NA	17.1	NA	.815^a^
Smoker (current)	4/52	7.7	4/52	7.7	6/57	10.5	.830
Mother smoking (ever)	17/52	32.7	18/52	34.6	16/57	28.1	.754
Father smoking (ever)	16/52	30.8	24/52	46.2	25/57	43.9	.233
Passive smoking (at age 17)	24/52	46.2	30/52	57.7	32/57	56.1	.441
Furry pets (current)	30/52	57.7	29/52	55.8	34/57	59.6	.902
Upper secondary school	34/43	79.1	28/35	80.0	47/51	92.2	.124
Vocational school	9/43	20.9	6/35	17.1	4/51	7.8	.124
Not in school	0/43	0.0	1/35	2.9	0/51	0.0	.124
Probiotics weekly or daily in past 12 mo	11/52	21.2	9/52	17.3	9/57	15.8	.815
Probiotics during last 3 y	35/52	67.3	37/52	71.2	41/57	71.9	.907

*Note*: Categorial variables analysed by *χ* ^2^ test. *P* < .05 indicated in bold.

Abbreviations: AE/CMA+, patients with atopic eczema and confirmed cows’ milk allergy in infancy; AE/CMA−, patients with atopic eczema and excluded cows’ milk allergy in infancy; ANOVA, analysis of variance.

^a^ Analysis with ANOVA.

### 3.2 IgE‐mediated sensitisation

The AE/CMA+ group was most frequently sensitised to all tested allergens, followed by the AE/CMA− group, and the control group (Table 2). With a cut‐off level of 0.35 kU/L there was a significant difference in sensitisation between the AE/CMA+ group and the control group as regards horse (*P* = .019), cat (*P* = .003), dog (*P* = .014), egg white (*P* = .024) and wheat (*P* = .017) allergens, and for any food‐specific IgE (*P* = .001), while differences compared with the AE/CMA− group were not significant. For birch (*P* < .001), timothy (*P* = .008), for any inhalant‐specific IgE (*P* < .001) or for any specific IgE (*P* < .001) the differences were significant as regards both the AE/CMA+ group compared with the control group and the AE/CMA− group compared with the control group. However, when the cut‐off level was set at 0.7 kU/L, the AE/CMA+ group was significantly more sensitised to any food‐specific IgE compared with both the AE/CMA− group and the controls (*P* = .003). We conducted a separate analysis of original patients who were sensitised as infants (sensitisation defined as either a positive skin prick test (mean weal diameter ≥3 mm, greater than the negative control) or s‐IgE > 0.35 kU/L for any tested allergen), divided into two groups according to CMA status, to estimate the effect of CMA while controlling for sensitisation. In this subanalysis the group sizes were AE/CMA+, n = 27 and AE/CMA−, n = 22. No significant differences in sensitisation in adolescence were found when all patients had been sensitised in infancy.

**Table 2:** Proportions of sensitisation to food and inhalant allergens

	AE/CMA+ (n = 40)	AE/CMA− (n = 38)	Controls (n = 50)	
	%	%	%	*P* value
Horse	47.5	28.9	20.0	**.019** *
Cat	70.0	52.6	34.0	**.003** *
Dog	62.5	50.0	32.0	**.014** *
Birch	80.0	65.8	40.0	**<.001** **
Mugwort	57.5	44.7	18.0	**<.001** **
Timothy	65.0	63.2	36.0	**.008** **
Wheat	37.5	26.3	12.0	**.017** *
Milk	15.0	2.6	4.0	.107
Egg white	22.5	11.7	4.0	**.024** *
Any food	50.0	28.9	14.0	**.001** *
Any inhalant	90.0	73.7	48.0	**<.001** **
Any	90.0	73.7	48.0	**<.001** **

*Note*: Sensitisation was defined as s‐IgE > 0.35 kU/L. Group comparisons by *χ* ^2^ test. *P* < .05 indicated in bold.

Abbreviations: AE/CMA+, patients with atopic eczema and confirmed cows’ milk allergy in infancy; AE/CMA−, patients with atopic eczema and excluded cows' milk allergy in infancy; IgE, immunoglobulin E.

* Significant difference: AE/CMA+ vs control.

** Significant difference: AE/CMA+ vs control and AE/CMA− vs control.

### 3.3 Allergic symptoms based on the ISAAC questionnaire

On the basis of ISAAC questionnaire data, the AE/CMA+ group reported a significantly higher lifetime prevalence of wheezing compared with the AE/CMA− group or the control group (*P* = .001) (Table 3). The AE/CMA+ group also reported a significantly higher lifetime prevalence of rhinitis compared with both the AE/CMA− group and the control group (*P* = .004). Additionally, the AE/CMA+ group reported a significantly higher lifetime prevalence of hay fever compared with the AE/CMA− group or the control group (*P* < .001). Both AE/CMA+ and the AE/CMA− groups reported significantly more “interference of rhinitis with daily activities in the past 12 months” (moderately or a lot compared with a little or none) compared with the control group (*P* = .006). Both groups also reported a significantly higher lifetime prevalence of an itchy rash (*P* = .006), and eczema at any time (*P* < .001) compared with the control group. In the AE/CMA+ group, compared with the controls, both ISAAC‐based current rhinoconjunctivitis (*P* = .003) and ISAAC‐based current eczema (*P* = .003) were significantly more frequent. Table 3 shows a breakdown of proportions for all ISAAC questions. We carried out a subanalysis of original patients who were sensitised during infancy, measured by PRICK and/or IgE, to further investigate the effect of CMA, and control for sensitisation. The patients were divided in two groups according to CMA status; group sizes were AE/CMA+, n = 27 and AE/CMA−, n = 12. There were no significant differences in reported symptoms. Compared with the control group, AE with CMA was associated with increased risks of wheezing (aOR, 4.77), rhinitis (aOR, 6.22), rhinitis symptoms in the past 12 months (aOR, 18.93), hay fever (aOR, 8.94) and “interference of rhinitis with daily activities in the past 12 months” (aOR, 9.39) (Table 4). Atopic eczema with a suspicion of CMA, compared with the control group, was associated with hay fever (aOR, 2.73) and “interference of rhinitis with daily activities in the past 12 months” (aOR, 7.65). In the analysis of combined ISAAC questions, AE with CMA, as well as AE with a suspicion of CMA were associated with current rhinoconjunctivitis (aOR, 6.00 and 3.42, respectively) and with current eczema (aOR, 6.08 and 2.99, respectively).

**Table 3:** Comparison of proportions of allergic symptoms from ISAAC core questionnaire responses

	AE/CMA+		AE/CMA−		Controls		
	n	%	n	%	n	%	*P* value
Wheezing ever	33/52	63.5	18/52	34.6	18/57	31.6	**.001** *
Wheezing 12 mo	17/33	51.5	11/19	57.9	9/18	50.0	.906
Wheezing attacks 12 mo (4 or more)	8/17	47.1	6/12	50.0	2/9	22.2	.447
Night wheezing 12 mo (ever)	5/17	29.4	3/12	25.0	2/9	22.2	1.000
Limited speaking 12 mo	5/17	29.4	4/12	33.3	2/9	22.2	.905
Asthma ever	14/52	26.9	9/52	17.3	8/57	14.0	.218
Exercise wheezing 12 mo	16/52	30.8	13/52	25.0	10/57	17.5	.279
Night cough 12 mo	12/52	23.1	8/52	15.4	10/57	17.5	.588
Rhinitis ever	44/52	84.6	32/52	61.5	32/57	56.1	**.004** *
Rhinitis 12 mo	42/44	95.5	28/34	82.4	25/32	78.1	.052
Itchy‐watery eyes with rhinitis 12 mo	28/42	66.7	22/28	78.6	13/25	52.0	.133
Rhinitis interference with daily activities 12 mo^a^	19/42	45.2	10/28	35.7	2/25	8.0	**.006** **
Hay fever ever	40/52	76.9	27/52	51.9	19/57	33.3	**<.001** *
Itchy rash ever	38/52	73.1	28/51	54.9	14/57	24.6	**<.001** **
Itchy rash 12 mo	31/38	81.6	17/28	60.7	11/14	78.6	.169
Itchy rash skin folds ever	25/31	80.6	17/17	100.0	10/11	90.9	.105
Itchy rash cleared 12 mo	17/31	54.8	12/17	70.6	7/11	63.6	.597
Itchy rash kept awake 12 mo (ever)	12/31	38.7	8/17	47.1	6/11	54.5	.647
Eczema ever	43/52	82.7	39/51	76.5	21/57	36.8	**<.001** **

Abbreviations: AE/CMA+, patients with atopic eczema and confirmed cows' milk allergy in infancy; AE/CMA−, patients with atopic eczema and excluded cows' milk allergy in infancy; ISAAC, International Study of Asthma and Allergies in Childhood.

^a^ Moderate amount or a lot.

* Significant difference: AE/CMA+ vs AE/CMA−, and AE/CMA+ vs controls.

** Significant difference: AE/CMA+ vs controls, and AE/CMA− vs controls.

**Table 4:** Association between CMA status in infancy and adolescent allergic symptoms from ISAAC core questionnaire responses

	Unadjusted OR (95% CI)		Adjusted OR* (95% CI)	
	AE/CMA+ (n = 52)	AE/CMA− (n = 52)	AE/CMA+ (n = 52)	AE/CMA− (n = 52)
Wheezing ever	**3.76 (1.70‐8.33)**	1.15 (0.52‐2.55)	**4.77 (2.01‐11.30)**	1.41 (0.60‐3.28)
Rhinitis ever	**4.30 (1.72‐10.75)**	1.25 (0.58‐2.69)	**6.22 (2.25‐17.24)**	1.64 (0.71‐3.83)
Rhinitis 12 mo	**5.88 (1.13‐30.54)**	1.31 (0.39‐4.41)	**18.93 (2.53‐141.60)**	3.52 (0.72‐17.33)
Rhinitis interference with daily activities 12 mo^a^	**9.50 (1.98‐45.55)**	**6.39 (1.24‐32.89)**	**9.39 (1.81‐48.56)**	**7.65 (1.30‐44.97)**
Hay fever ever	**6.67 (2.86‐15.57)**	2.16 (0.996‐4.68)	**8.94 (3.49‐22.90)**	**2.73 (1.17‐6.36)**
Itchy rash ever	**8.34 (3.53‐19.70)**	**3.74 (1.65‐8.47)**	**11.60 (4.45‐30.21)**	**4.68 (1.93‐11.35)**
Eczema ever	**8.19 (3.34‐20.10)**	**5.57 (2.40‐12.93)**	**9.13 (3.48‐23.92)**	**6.14 (2.49‐15.17)**
ISAAC‐based current rhinoconjunctivitis	**3.95 (1.73‐9.01)**	**2.48 (1.09‐5.68)**	**6.00 (2.40‐15.06)**	**3.42 (1.39‐8.43)**
ISAAC‐based current eczema	**4.35 (1.82‐10.42)**	2.28 (0.93‐5.59)	**6.08 (2.37‐15.63)**	**2.99 (1.16‐7.72)**

Abbreviations: AE/CMA+, patients with atopic eczema and confirmed cows’ milk allergy in infancy; AE/CMA−, patients with atopic eczema and excluded cows’ milk allergy in infancy; CI, confidence interval; ISSAC, International Study of Asthma and Allergies in Childhood; OR, odds ratio.

^a^ “A moderate amount” or “a lot” compared with “not at all” or “a little”.

* Adjusted for sex, pets, and passive smoking. *P* < .05 indicated in bold. The control group is the reference group.

Original patients were analysed separately to adjust for the effect of AE and demographic characteristics collected only in the original study. This also enabled us to compare the effect of CMA with the effect of any atopic sensitisation in infancy. Compared with AE and a suspicion of CMA, AE plus CMA was associated with increased risks of wheezing (crude odds ratio [cOR], 2.81, 95% CI, 1.47‐7.32; aOR, 3.80, 95% CI, 1.59‐9.06), rhinitis (cOR, 3.44, 95% CI, 1.35‐8.78; aOR, 2.90, 95% CI, 1.16‐7.21) and hay fever (cOR, 3.09; 95% CI, 1.33‐7.18; aOR, 3.32, 95% CI, 1.25‐8.87). Sensitisation in infancy, measured by PRICK and/or IgE, was significantly associated with an increased risk of rhinitis (aOR, 4.60; 95% CI, 1.82‐11.63), but not with wheezing (aOR, 1.00; 95% CI, 0.41‐2.47) or hay fever (aOR 2.32; 95% CI, 0.89‐6.06).

### 3.4 Doctor‐diagnosed allergic disease

Both the AE/CMA+ group and the AE/CMA− group (compared with the control group) more frequently reported a doctor's diagnosis of rhinoconjunctivitis (50%, 46.2% and 21.1%, respectively). Proportions differed significantly between all groups as regards reported food allergy (100%, 53.8% and 10.5%, respectively). The AE/CMA+ group showed a significant association with rhinoconjunctivitis (aOR 3.21, 95% CI, 1.39‐7.43). Differences between groups were not significant as regards a doctor's diagnosis of asthma (26.9%, 17.3% and 12.3%, respectively).

### 3.5 Atopic eczema activity by SCORAD scores and lung function

The severity of AE measured in terms of SCORAD scores was highest in the AE/CMA+ group (mean 8.8, median 5.0), second highest in the AE/CMA− group (mean 6.0, median 0.10) and lowest in the control group (mean 3.87, median 0.0). The difference between groups was significant as regards the AE/CMA+ group compared with the control group (*P* = .022). Spirometry results are shown in Table 5.

**Table 5:** Spirometry results (percentages) in patients with atopic eczema and cows’ milk allergy in infancy, atopic eczema and a suspicion of cows’ milk allergy in infancy, and controls

	AE/CMA+ (n = 42)		AE/CMA− (n = 36)		Controls (n = 44)		
	Mean	Adjusted mean	Mean	Adjusted mean	Mean	Adjusted mean	*P* value
FVC	98.9	98.1	97.7	97.8	94.3	95.1	.209^a^
FEV1	96.3	95.2	93.4	93.3	90.6	91.7	.270^a^
FEV1/FVC	88.5		88.2		90.0		.49^b^

*Note*: Among 130 spirometry tests performed, 122 were of adequate quality to be included in the analysis. The same parameters were evaluated after bronchodilation; differences remained nonsignificant (data not shown)

Abbreviations: AE/CMA+, patients with atopic eczema and confirmed cows’ milk allergy in infancy; AE/CMA−, patients with atopic eczema and excluded cows’ milk allergy in infancy; ANCOVA, analysis of covariance; ANOVA, analysis of variance; FVC, forced vital capacity; FEV1, forced expiratory volume in one second.

^a^ ANCOVA, means adjusted for sex and cooperation.

^b^ ANOVA, no covariates met the assumptions for ANCOVA.

### 3.6 Natural history of CMA

Among the 52 patients who were diagnosed with CMA in the original study, 44 had since included cows' milk in their diet. For seven patients this data was missing. Thirty patients disclosed the age at which milk had been reintroduced in their diet. The mean age at this inclusion was 5.5 years. Five patients had received oral immunotherapy for CMA. One patient, who had not received oral immunotherapy, reported that their IgE‐mediated CMA was still ongoing. Among the 45 original CMA patients whose current allergy status was known, IgE for cows' milk was measured in 36. With a 0.35 kU/L cut‐off value, four of 35 who were classified as tolerant were still sensitised. With a 0.7 kU/L cut‐off value, this figure was two of 35. The one patient who reported ongoing CMA was not sensitised on the basis of either cut‐off value.

## 4 DISCUSSION

In this study we examined sensitisation, allergic symptoms, severity of atopic eczema, lung function and diagnoses of allergic disease in adolescence among patients that in infancy had AE with and without CMA, and unselected controls. Allergic sensitisation to the tested allergens was most frequent in the AE/CMA+ group. The differences between groups were mainly significant between the AE/CMA+ group vs the control group, but at an IgE cut‐off level of 0.7 kU/L a difference between the AE/CMA+ group compared with the AE/CMA− group was observed in connection with sensitisation to any food allergen. Among allergic symptoms the AE/CMA+ group reported a significantly higher lifetime prevalence of wheezing, rhinitis and hay fever compared with patients with infantile eczema only, and controls. However, the AE/CMA+ group compared with the AE/CMA− group were not significantly more often diagnosed with asthma or allergic rhinoconjunctivitis.

Most studies on CMA have been focused on infants and younger children, whereas studies on adolescents are infrequent; thus our study provides novel information. In our study, the original diagnosis of CMA relied on a DBPCFC, which is a major strength, as in previous studies on adolescents and young adults, results based on sensitisation or open food challenges have been reported.^14^, ^19^ The use of a standardised, validated questionnaire for allergic symptoms facilitates comparison with other studies. A drawback of this study is the unequal sex distribution, as it is known that differences in allergic disease vary between sexes as age increases.^20^ We believe the majority of control patients were female, because most of these patients attended upper secondary school, where the majority of students are female. Sex and educational level elevate the likelihood to participate in scientific studies, which was accentuated in our adolescent population. In addition, allergic diagnoses at follow‐up were not verified from patient records, but relied on patient reporting only. In some analyses the group sizes were so small that there may be a risk of a type II error, which makes it difficult to draw any firm conclusions. It is noteworthy that our group of CMA patients included both IgE‐mediated and non–IgE‐mediated allergy.

In our study there was a significant difference in sensitisation at the cut‐off level 0.35 kU/L between the AE/CMA+ group and the control group for all tested allergens, except, interestingly, cows' milk. An explanation could be that sensitisation to cows' milk has usually declined by the time of adolescence, even in previously allergic patients.^21^ A similar difference in sensitisation patterns has previously been found in a prospective study from Finland in which 118 children with CMA were followed up to the age of 8.6 years.^22^ Sensitisation was also more frequent in the AE/CMA+ group compared with the AE/CMA− group for all allergens, though this difference was not significant. However, we also carried out an analysis with a cut‐off of 0.7 kU/L, as some patients with s‐IgE levels below this may not be truly sensitised. In this analysis a significant difference in sensitisation to any food‐specific IgE was observed between the AE/CMA+ group and the AE/CMA− group. Even in adolescence CMA patients are more sensitised to food allergens than patients with infantile eczema only. Analysis of the AE/CMA+ group and the AE/CMA− group that included only patients that were sensitised as infants showed no difference in current sensitisation. It may be that the observed difference between the three groups is thus more attributable to the original IgE sensitisation than CMA itself, but the number of patients in the subanalysis was too small to draw any firm conclusions. Previous studies support the idea that non–IgE‐mediated CMA is a benign condition that is not associated with an increased risk of atopic diseases.^23^

We observed that subjects with AE and CMA in infancy, compared with those with AE only, and unselected controls, had a higher lifetime prevalence of certain allergic symptoms: wheeze, noninfectious rhinitis and hay fever. For wheeze and noninfectious rhinitis, as well as rhinitis symptoms in the past 12 months, the associated aOR was significantly higher in the AE/CMA+ group compared with controls, but not in the AE/CMA− group compared with controls. Subjects with infantile AE both with and without CMA showed an association with ISAAC‐based diagnosis of current rhinoconjunctivitis and ISAAC‐based diagnosis of current eczema compared with controls, and this association seemed stronger in the AE/CMA+ group. Thus, patients with CMA not only exhibited more allergic symptoms in adolescence, but the symptoms were also more severe. We also analysed the AE/CMA+ group and AE/CMA− group in a separate regression model, as this allowed us to adjust for confounders that had been examined in the original study, and to examine the effect of any sensitisation compared with clinical CMA. The odds of wheezing, noninfectious rhinitis and hay fever were higher in the AE/CMA+ group compared with the AE/CMA− group. This adjusted model also showed an association between early sensitisation and rhinitis, but not hay fever. These results are somewhat contradictory, which indicates that early sensitisation, as well as clinical CMA, has a separate influence on the exhibited atopic phenotype. However, the sample size was not large enough to draw any firm conclusions.

Asthma developed more frequently in the AE/CMA+ group than in the AE/CMA− or control group. Even though this difference was not significant, a higher prevalence of asthma in CMA patients vs controls has previously been observed.^22^ In our study, lung function did not differ between patient groups. Another study revealed that children with CMA in infancy, compared with controls, at an average age of 8.6 years showed more signs of airway inflammation, expressed as elevated fractional exhaled NO and more pronounced bronchial responsiveness to histamine.^24^ In a recently published study, it was found that sensitisation to cows' milk, hens' eggs or peanuts at the ages of 6 and 12 months, was associated with reduced FEV1 in adolescence, but the investigators did not analyse the individual effects of separate allergens, and the study was based on sensitisation and not diagnosed clinical allergy.^25^

Responses to ISAAC questions on AE (prevalence of rash ever, eczema ever and current eczema) showed significant differences in both original patient groups compared with the controls. This was expected, as AE was an inclusion criterion in the original study. This supports the notion that the ISAAC questionnaire was efficient in identifying symptoms correctly in our population. Despite not reaching statistical significance, in adolescence, there were more symptoms of eczema in the AE/CMA+ group than in the AE/CMA− group.

In a previous study, it was reported that among 139 CMA patients there was a tolerance acquisition rate of 54.6% by the age of five and 69% by the age of 15.^14^ Tolerance acquisition at a young age was similar in our study, the median age at acquisition being 5.5 years. However, the rate of acquisition of tolerance by the time of the follow‐up visit was 97.8% in our study, which was higher than the previously reported rate in adolescence. This may be because five patients in our study had received oral immunotherapy for CMA. By comparison, in another study among 39 infants with CMA, the acquisition of tolerance was 92% at 5 years and 97% at 15 years.^2^ Before 10 years age, 41% developed asthma, and 31% rhinoconjunctivitis. This risk was significantly increased in patients with IgE‐mediated allergy compared to patients with non–IgE‐mediated allergy. The CMA diagnosis in both studies was based on an open food challenge, and both IgE‐ and non–IgE‐mediated cases were included. The prevalence of rhinoconjunctivitis in our study was 50%, which was higher than in the above two studies and this may be explained by the fact that all our patients with CMA also had a diagnosis of AE. The study population affects the rate of acquisition of tolerance, which is higher in population based studies compared to studies from tertiary care centres that focus on patients with suspected or confirmed allergic disease.^6^, ^14^

Our findings supported the notion that CMA in infancy has implications on allergy‐related morbidity later in life, and it poses an independent risk of allergic symptoms, compared with eczema only. A recent study on single‐nucleotide polymorphism profiles and genetic predisposition to other diseases in patients with CMA indicated that there is an overlap in the genetic aetiology of CMA and asthma, but for CMA and AE, there is not.^26^ Our clinical results support this finding. To our knowledge, ours may be the first study carried out to compare patients with CMA and AE with patients with AE only.

## CONFLICT OF INTERESTS

The authors declare that there are no conflict of interests.

## Supporting information

Supporting information

Supporting information

## Data Availability

The data that support the findings of this study are available from the corresponding author upon reasonable request.
