# Supplementary figures and images for: Allergic symptoms and sensitisation in adolescents with cows' milk allergy and atopic eczema in infancy
Source: Immun Inflamm Dis. 2020 Jun 21;8(3):423–33. doi: 10.1002/iid3.324 (PMC7416017; doi:10.1002/iid3.324)

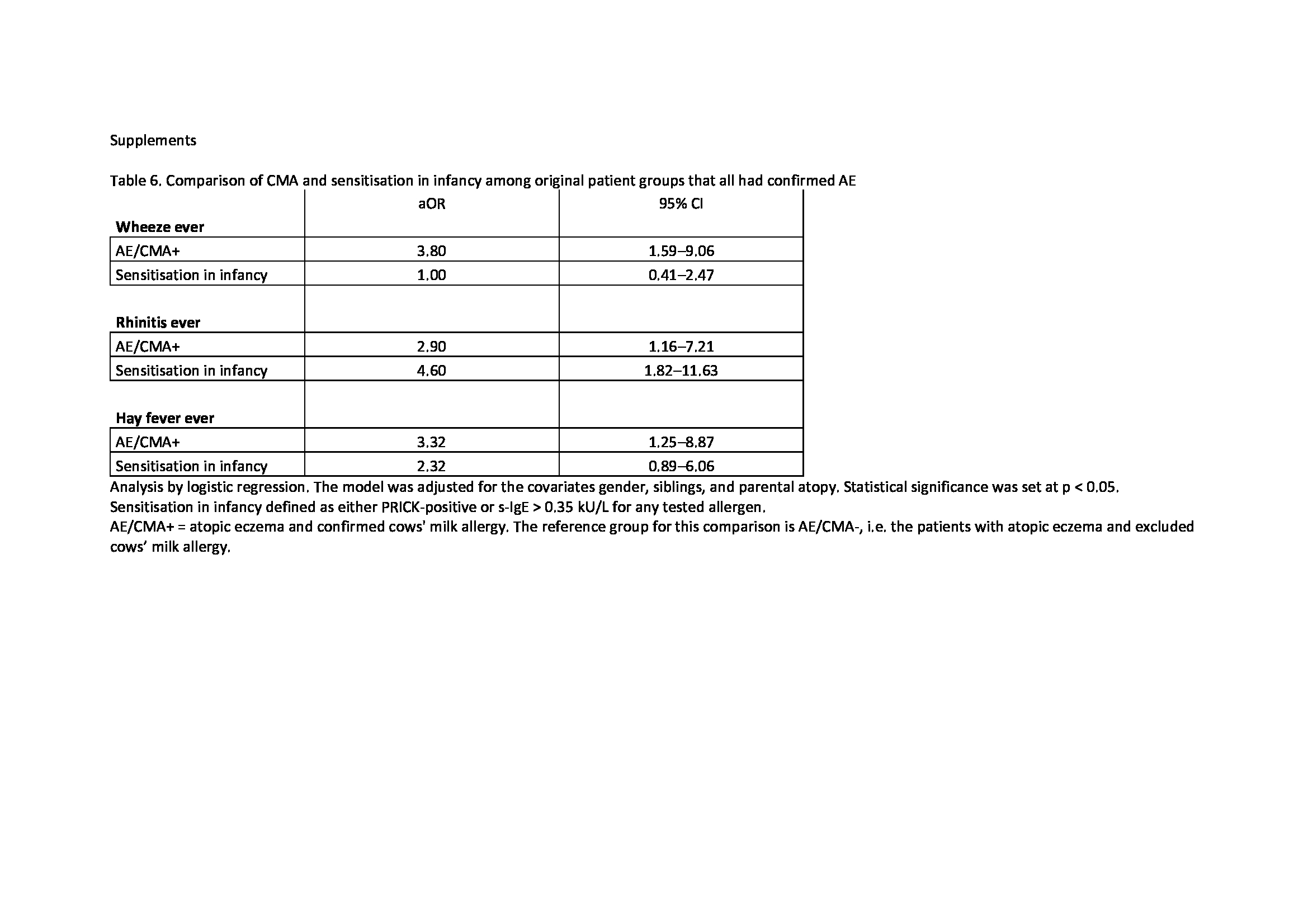

Supplement: Supplementary file 1 — Supporting information [file IID3-8-423-s001.jpeg]
